# Inter-Species Investigation of Biological Traits among Eight *Echinochloa* Species

**DOI:** 10.3390/plants12173085

**Published:** 2023-08-28

**Authors:** Xuli Hu, Runqiang Liu, Honghao Mao, Yong Xu, Bin Chen, Yongfeng Li, Xia Yang

**Affiliations:** 1 Provincial Key Laboratory of Agrobiology, Jiangsu Academy of Agricultural Sciences, Nanjing 210014, China; 2 Zhongshan Biological Breeding Laboratory, Nanjing 210014, China; 3 Henan Institute of Science and Technology, Xinxiang 453003, China

**Keywords:** *Ehcinochloa* spp., biological properties, different sowing dates, whole growth period, photosynthetic rates

## Abstract

Due to the diversity of *Echinochloa* species and the limited understanding of their damage processes in rice fields, clarifying the biological properties of distinct species could help create a foundation for effective control techniques. Pot experiments and field competition trials were conducted using eight *Echinochloa* species to elucidate their biological differences and assess their varying levels of negative impact on rice. The survey outcomes showed that *E. oryzoides* had the highest 1000-grain weight (3.12 g) while *E. colona* had the lowest (0.90 g). The largest grain number per spikelet found in *E. glabrescens* (940) was 3.4 times greater than that in *E. oryzoides* (277). Different species responded variably to changes in temperature and photoperiod. Except for *E. caudate*, all *Echinochloa* species exhibited a shortened growth period with the delay of the sowing date. Under field competitive conditions, all *Echinochloa* species exhibited significantly greater net photosynthetic rates than rice, with *E. crusgalli* exhibiting the highest photosynthetic capacity. Moreover, in this resource-limited setting, barnyardgrass species had a decrease in tiller formation and panicle initiation but a significant increase in plant height. These findings contribute valuable insights into the biological characteristics of barnyardgrass populations and provide guidance for implementing effective control measures in rice fields.

## 1. Introduction

In various rice-producing regions of China, barnyardgrass is a widespread and difficult-to-control weed with broad ecological tolerance, high seed reproductive capacity, and significant negative impacts on crop production [1]. As a C4 plant, barnyardgrass competes with crops for resources such as water, nutrients, space, and sunlight in the field, making it a major limiting factor for efficient agricultural production [2,3]. Barnyardgrass has the ability to mimic crops, and once it goes undetected in rice fields, crop yield reduction becomes inevitable [4]. It is estimated that crop losses due to weed infestation in China exceed three million tons annually, with barnyardgrass being a significant contributor to these losses [5]. Herbicides have been the primary means of weed control in agricultural fields but the excessive use of herbicides targeting a single site of action has led to the emergence and increasing trend of barnyardgrass herbicide resistance in multiple rice cultivation areas [6,7]. Moreover, improper herbicide use can result in pesticide residue, soil compaction, and agricultural nonpoint source pollution. Therefore, comprehensive weed management and a reduction in herbicide usage are urgently needed, necessitating detailed research on the biological characteristics of weeds.

The English revised edition of the Flora of China (FOC, http://www.iplant.cn/foc (accessed on 10 July 2023)) reports that eight species of the *Echinochloa* genus, commonly referred to as barnyardgrass, are found in China. Among them, six species pose a threat to rice crops, including *E. crusgalli*, *E. glabrescens*, *E. oryzoides*, *E. colona*, *E. cruspavonis,* and *E. caudate* [8]. The species *E. crusgalli* further includes six subspecies: *E. crusgalli* (original variant), *E. zelayensis*, *E. mitis*, *E. crusgalli* var. *austro-japonensis*, *E. crusgalli* var. *breviseta,* and *E. crusgalli* var. *praticola* Ohwi. Each species causes different levels of damage to crops and significant interspecific differences may affect the effectiveness of chemical or biological control methods which depend on the habits and biological variations of barnyardgrass. The morphological differences among different species can be distinguished based on the characteristics of their spikelets. Chen et al. (2019) and Lu et al. (2014) [8,9] conducted a species identification and distribution survey of barnyardgrass in multiple provinces across China. The primary morphological characteristics of each species can be found at the following web addresses: http://www.iplant.cn/info/Echinochloa?t=foc and https://www.zhiwutong.com/latin/Gramineae/Echinochloa.htm (accessed on 10 July 2023).

Different species of *Echinochloa* have varying effects on crop yield reduction. Competition from approximately 25 *E. crusgalli* plants per square meter can lead to approximately 70% yield loss [10]. Zhang et al. (2021) [5] found that the coexistence of *E. mitis*, *E. zelayensis,* and *E. colona* significantly inhibited the aboveground growth of rice during the heading stage, resulting in yield losses of 10.6–46.5%. Moreover, barnyardgrass interferes with rice photosynthetic rates and dry matter accumulation during the grain-filling stage, thus causing a decline in grain yield and quality [11]. For every 10% increase in barnyardgrass control, the spike density of rice increases by 14 spikes/m^2^, resulting in an increase in yield of 750 kg per hectare [12]. When rice and barnyardgrass compete under limited resource conditions, barnyardgrass may increase its stem length to avoid shading by the crop, while rice interference reduces the inflorescence and aboveground biomass of barnyardgrass. In contrast, barnyardgrass mitigates rice interference by increasing its leaf weight ratio [13]. Tang et al. (2009) [14] found that the occurrence of barnyardgrass in the field and the use of herbicides can be significantly reduced by utilizing mixed planting of multiple rice varieties to increase genetic diversity. Wu et al. (2010) [15] demonstrated that increasing the planting density of sorghum to 7.5 plants/m^2^ reduced the density, biomass, and seed production of the weed species *E. esculenta* by 22%, 27%, and 38%, respectively. In conclusion, crop competitiveness is a complex attribute involving the ability of crops to maintain growth and yield under weed interference (weed tolerance) and to suppress weed growth through competition [16].

Even if individual species of barnyardgrass may have similar morphological characteristics, they can exhibit differences in their response to herbicides [2]. These interspecies differences can impact their competitive interactions with crops, potentially influencing the effectiveness of chemical or agronomic measures for weed control. Previous studies have often focused on specific species of barnyardgrass under specific environmental conditions, rarely comparing multiple species of barnyardgrass under equivalent conditions. In this study, an experiment was conducted to investigate the physiological and biological traits of eight *Echinochloa* species under three sowing periods and assess their varying levels of negative impact on rice. The aim of this research is to reveal the variations in interspecies biological traits among barnyardgrass species and to clarify the factors contributing to the competitive disadvantage of rice in the field. The findings will hold great significance for biological research and the scientific management of barnyardgrass in Chinese paddy fields.

## 2. Results

### 2.1. Differences in 1000-Grain Weight and Spikelet Number among Barnyardgrass Species

**Figure 1:**
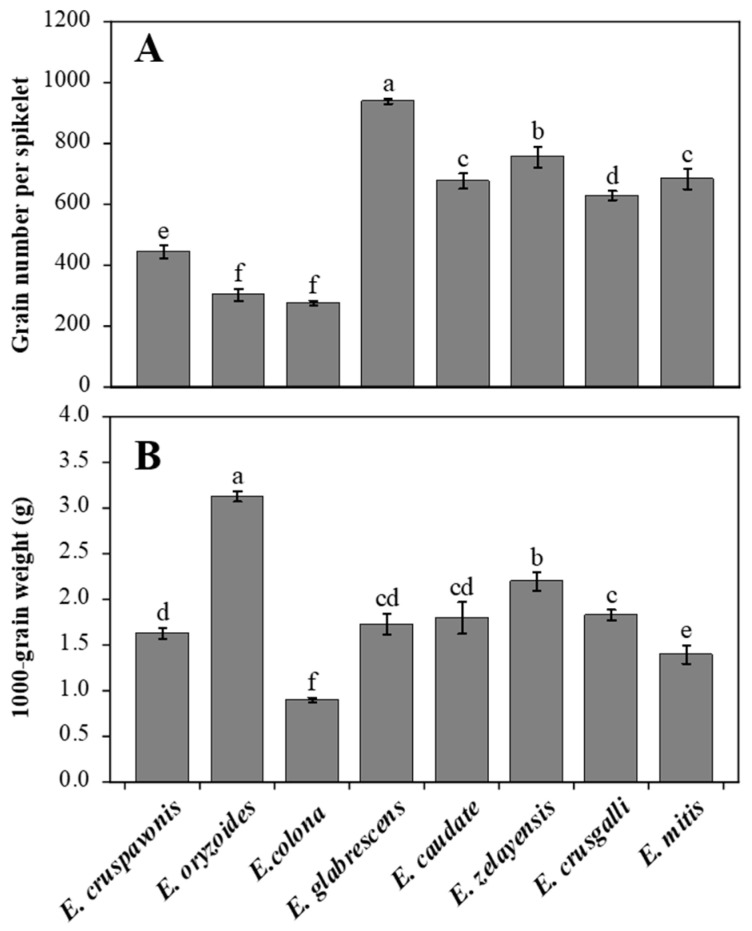
Spikelet grain number (**A**) and 1000-grain weight (**B**) of different barnyardgrass species. Vertical bars are standard errors (*n* = 6). a–f: Different letters indicate significant differences in spikelet grain number or 1000-grain weight among different barnyardgrass species using Duncan’s multiple range test (*p* ≤ 0.05).

For gramineous crops, a higher 1000-grain weight has been associated with an improved germination rate and certain agronomic traits [17]. It remains unclear whether the same relationship holds true for barnyardgrass. Therefore, elucidating the variation in 1000-grain weight among different barnyardgrass species is crucial for further investigating the biological characteristics of seed germination. There were significant differences in the 1000-grain weight among the barnyardgrass species (Figure 1B). *E. oryzoides* had the highest 1000-grain weight (3.13 g), which was significantly higher than that of the other barnyardgrass species. *E. glabrescens* (1.73 g), *E. caudate* (1.80 g), and *E. crusgalli* (1.83 g) had similar 1000-grain weights with no significant differences. *E. cruspavonis* (1.63 g) had a higher 1000-grain weight than *E. mitis* (1.40 g). The 1000-grain weight of *E. colona* was the lowest at only 0.90 g. *E. oryzoides* had a 1000-grain weight approximately 2.2 times that of *E. mitis* and 3.5 times that of *E. colona*. 

The seed yield of barnyardgrass is mainly determined by the effective spike number, number of grains per spike, and grain weight. The number of grains per spike is a key factor in spike grain formation, directly affecting the number of grains and grain yield. We also investigated the grain number per spikelet (Figure 1A) which was closely associated with the reproductive coefficient of barnyardgrass [18]. *E. glabrescens* had the highest number of grains per spike, reaching 940 grains, followed by *E. zelayensis* with 757 grains. There were no significant differences in the number of grains per spike between *E. mitis* and *E. caudate*, which was approximately 680 grains. *E. oryzoides* and *E. colona* had the lowest numbers of grains per spike, with both not exceeding 350 grains. The number of grains per spike in *E. glabrescens* was 3.1 times greater than that in *E. oryzoides* and 3.4 times greater than that in *E. colona*. It is worth noting that *E. oryzoides* had the highest 1000-grain weight but the lowest number of grains per spike. The 1000-grain weight and number of grains per spike of *E. colona* were the lowest among all barnyardgrass species.

### 2.2. Number of Tillers and Panicles of Barnyardgrass Species under Different Sowing Conditions 

Temperature and photoperiod are important variables controlling phenological development in weeds [19]. A pot experiment was conducted to study the response of barnyardgrass phenological development to changes in temperature and photoperiods by sowing barnyardgrass in three batches, each with different ecological conditions (temperature and photoperiod).

**Figure 2:**
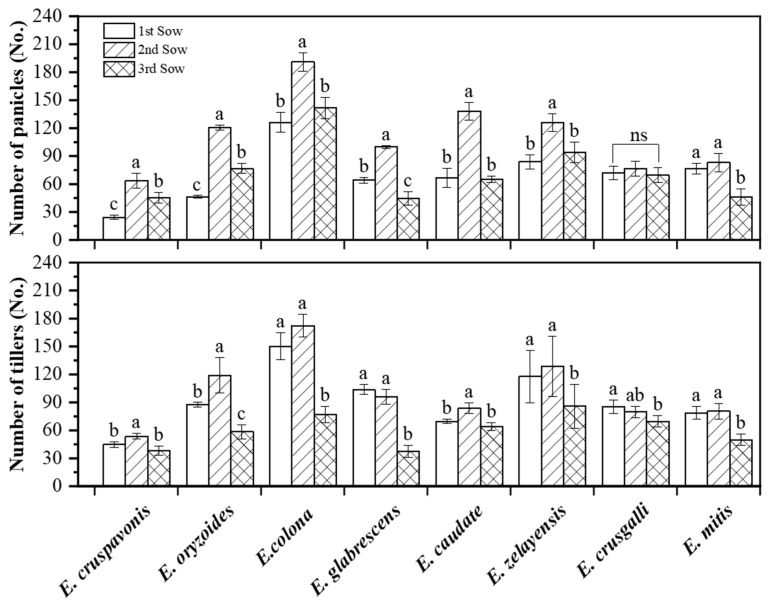
Comparison of the number of tillers and panicles of eight barnyardgrass species under three sowing periods. The “1st sow”, “2nd sow”, and “3rd sow” refer to the first sowing period on 24 April, the second on 7 June, and the third on 26 June, respectively. Vertical bars are standard errors (*n* = 6). “a–c” represents the significance levels (*p* ≤ 0.05) determined using Duncan’s multiple range test.

The three sowing conditions had a range of influences on the tiller and panicle numbers of barnyardgrass species (Figure 2). Among the different sowing conditions, *E. cruspavonis* displayed the highest number of tillers under the second sowing condition, with 53 tillers, which was significantly greater than the number observed under the other two sowing conditions. *E. caudate* maintained a similar trend to that of *E. cruspavonis* in this study. *E. oryzoides* showed significant differences in the number of tillers among the three sowing conditions, with 119 tillers in the first sowing condition, 87 tillers in the second, and only 58 tillers in the third. *E. colona* had the highest number of tillers under the second sowing condition (172), which was slightly higher than that under the first sowing condition (150) but significantly higher than that under the third sowing condition (77). *E. glabrescens*, *E. zelayensis*, *E. mitis*, and *E. crusgalli* showed a similar trend to *E. colona*, with significantly higher tiller numbers in the first sowing condition compared to the third sowing condition. Overall, *E. colona* exhibited the strongest tillering ability (mean of 133 tillers), while *E. cruspavonis* had the weakest tillering ability (mean of 46), with a fold difference of 2.9.

Among the three sowing periods, all species had the highest numbers of panicles under the second sowing condition (Figure 2). *E. cruspavonis* had 64 panicles under the second sowing condition, 45 panicles under the third, and only 24 panicles under the first, with significant differences among the three sowing conditions. *E. oryzoides* showed a similar trend to *E. cruspavonis*. *E. colona* had 191 panicles under the second sowing condition, 126 panicles under the first, and 142 panicles under the third, with no significant differences in panicle numbers between the first and third sowing conditions. *E. caudate* and *E. zelayensis* exhibited a similar trend to *E. colona*. *E. glabrescens* showed significant differences in the number of panicles under the three sowing conditions, with panicle numbers of 100, 64, and 44, respectively. *E. mitis* showed no significant differences in panicle numbers between the first and second sowing conditions but a significant decrease under the third sowing condition, with only 46 panicles. *E. crusgalli* showed no significant differences in panicle numbers among different the sowing conditions. Panicle numbers are influenced by tiller numbers and they are positively correlated, indicating that more tillers result in more panicles.

### 2.3. Plant Height and Number of Leaves on the Main Stem of Barnyardgrass Species under Different Sowing Conditions 

**Figure 3:**
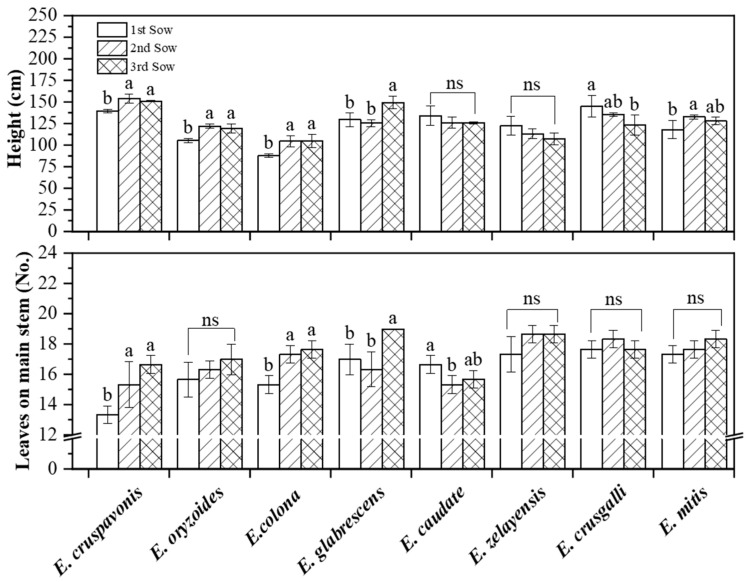
Plant height and number of leaves on the main stems of eight barnyardgrass species under three sowing periods. The “1st sow”, “2nd sow”, and “3rd sow” refer to the first sowing period on 24 April, the second on 7 June, and the third on 26 June, respectively. Vertical bars are standard errors (*n* = 6). “a, b” represents the significance levels (*p* ≤ 0.05) determined using Duncan’s multiple range test. The “ns” means no significant difference.

There may be a positive correlation between plant height and internode length in barnyardgrass species (Figure 3). In the second and third sowing periods, the plant heights of *E. cruspavonis* were 154.33 cm and 151.33 cm, respectively, which were not significantly different and but were significantly higher than the plant height of 140.00 cm in the first sowing period. *E. oryzoides*, *E. colona*, and *E. mitis* exhibited a similar trend to *E. cruspavonis*. *E. zelayensis* and *E. caudate* showed no significant differences in plant height among the different sowing conditions. *E. glabrescens* had the highest plant height of 149.67 cm under the third sowing condition, which was significantly higher than the plant height under the first sowing condition (129.67 cm) and the second sowing condition (125.67 cm). *E. crusgalli* showed no significant differences in plant height between the first and second sowing conditions (145.33 cm and 165.67 cm) but was significantly taller under the first and second than under the third sowing condition (123.33 cm). The plant height of *E. colona* was the lowest among the eight species and it did not exceed 110 cm in all three sowing periods.

The number of leaves on the main stem of the different barnyardgrass species under the three sowing conditions was recorded in this experiment (Figure 3). *E. cruspavonis* had 16 leaves under the third sowing condition, which was significantly higher than the 13 leaves under the second sowing condition. There was an increasing trend in leaf number as the sowing conditions progressed. *E. colona* exhibited a similar trend to *E. cruspavonis*. *E. glabrescens* had the highest number of leaves under the third sowing condition (19), which was significantly higher than the number of leaves under the first (17) and second (16) sowing conditions. In contrast, *E. caudate* had the highest number of main stem leaves under the first sowing condition (16), which was significantly higher than those under the second and third sowing conditions. There were no significant differences in the number of main stem leaves among *E. oryzoides*, *E. zelayensis*, *E. crusgalli*, and *E. mitis*.

### 2.4. Duration of the Growth Period

**Figure 4:**
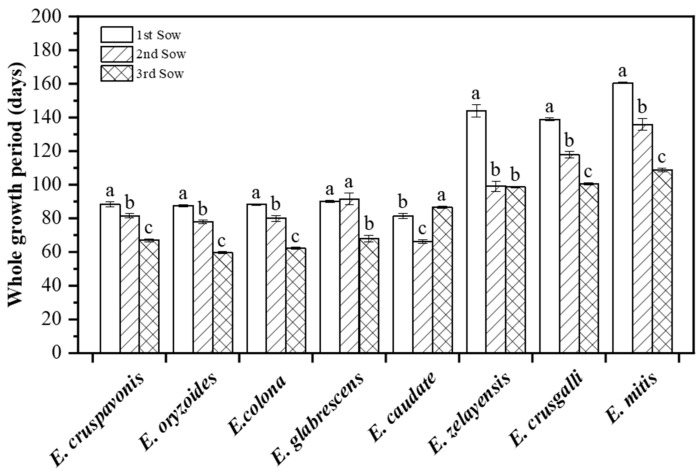
Whole growth days of different barnyardgrass species sown on different sowing dates. The “1st sow”, “2nd sow”, and “3rd sow” refer to the first sowing period on 24 April, the second on 7 June, and the third on 26 June, respectively. Vertical bars are standard errors (*n* = 6). “a–c” represents the significance levels (*p* ≤ 0.05) determined using Duncan’s multiple range test.

There were differences in the whole growth period times of the barnyardgrass species across the three sowing periods (Figure 4). *E. cruspavonis*, *E. oryzoides*, *E. colona*, *E. zelayensis*, *E. crusgalli*, and *E. mitis* demonstrated a trend of shorter growth periods under delayed seeding conditions. Except for *E. zelayensis* and *E. glabrescens*, the growth durations in days of the remaining barnyardgrass species exhibited significant variations under the three sowing conditions. *E. glabrescens* has similar growth periods in the first and second sowing conditions, with no significant differences. However, in the third sowing condition, the growth period of *E. glabrescens* was significantly shorter. In contrast, *E. caudate* had the longest growth period in the third sowing condition, reaching 86 d, which was significantly higher than that in the first (81 d) and second (66 d) sowing conditions. To summarize, *E. zelayensis*, *E. mitis*, and *E. crusgalli* had significantly longer growth periods, with average durations of 114 d, 119 d, and 135 d, respectively.

### 2.5. Differences in Photosynthesis between Rice and Barnyardgrass and Key Morphological Indicators under Field Competition

**Figure 5:**
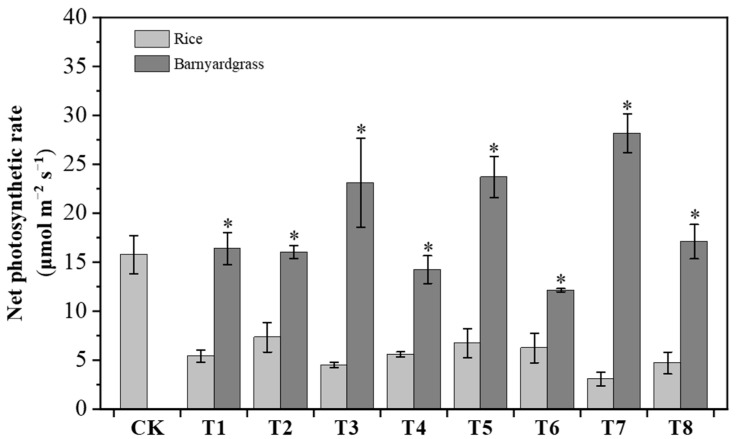
Net photosynthetic rates of rice and barnyardgrass under competitive conditions in the field. Control (CK): no barnyardgrass treatment; the barnyardgrass treatments T1, T2, T3, T4, T5, T6, T7, and T8 corresponded to *E. cruspavonis*, *E. oryzoides*, *E. colona*, *E. glabrescens*, *E. caudate*, *E. zelayensis*, *E. crusgalli*, and *E. mitis*, respectively. Error bars indicate standard errors calculated for three replications. Significant differences between samples were determined using a Student’s *t*-test. * Means are significantly different at 0.05 level.

During the tillering stage, we conducted measurements of the net photosynthetic rates of rice and barnyardgrass. The net photosynthetic rates of barnyardgrass were much higher than those of rice under different treatments (Figure 5). Compared to the control, the net photosynthetic rates of rice under competitive conditions were significantly suppressed. Rice seedlings with no barnyardgrass interference exhibited the highest net photosynthetic rate and the best growth. When different barnyardgrass treatments were applied, the photosynthesis of rice was significantly inhibited, and the degree of inhibition varied. *E. crusgalli* (T7) resulted in the most severe inhibition in rice, with the rice showing a net photosynthetic rate of only 19.5% compared to the control. In general, the net photosynthetic rate of rice in the treatment groups was less than half of that in the control group. There were variations in the photosynthetic rates among different barnyardgrass species. *E. crusgalli* (T7) had the highest photosynthetic rate among all barnyardgrass species, followed by *E. colona* (T3) and *E. caudate* (T5), which had similar levels of photosynthesis. *E. oryzoides* (T2), *E. cruspavonis* (T1), and *E. mitis* (T8) showed no significant differences in the photosynthetic rate. *E. zelayensis* (T6) had the lowest net photosynthetic rate at 12.17 µmol m^−2^ s^−1^ but it was still 1.86 times higher than that of the corresponding rice under competitive conditions. During the panicle initiation stage of barnyardgrass, over 80% of rice plants died in the field, making it impossible to measure subsequent photosynthetic parameters. This indicates that under the given density conditions, failure to timely control barnyardgrass can lead to the risk of crop loss.

We also assessed the morphological properties of barnyardgrass species under field competition conditions (Table 1). The tiller number is an important indicator of weed competitiveness. *E. colona* had the highest tiller number of approximately 48; that of *E. glabrescens* was significantly lower than that of *E. colona*, with approximately 32 tillers. *E. caudate*, *E. crusgalli*, and *E. mitis* showed no significant differences, with tiller numbers ranging from 18 to 23. *E. oryzoides* and *E. cruspavonis* had the fewest tillers, with 14 and 12, respectively. The tiller number of *E. colona* was 3.32 times that of *E. oryzoides* grass and 3.84 times that of *E. cruspavonis*.

*E. caudate*, *E. crusgalli*, and *E. mitis* had significantly greater plant heights than other barnyardgrass species, exceeding 200 cm. There were no significant differences in plant height among these three species. The following two species had similar plant heights with no discernible differences: *E. cruspavonis* (200.67 cm) and *E. zelayensis* (198.00 cm). *E. colona* and *E. glabrescens* did not show significant differences in plant height, with both being below 185.00 cm. *E. oryzoides* had the shortest plant height, reaching only 148.00 cm. There was a certain association between the number of internodes on the main stem and plant height. *E. crusgalli* had the highest number of internodes on the main stem, reaching up to 12. *E. mitis* had a slightly lower number of approximately 10. *E. cruspavonis*, *E. colona*, *E. glabrescens*, *E. caudate*, and *E. zelayensis* did not differ significantly in the number of internodes, which was between 8 and 9. *E. oryzoides* had the fewest internodes, with only 6. Generally, taller plants had a higher number of internodes. *E. oryzoides* had the shortest plant height and the fewest internodes among the studied species. Both *E. crusgalli* and *E. mitis* had more than 10 internodes, corresponding to plant heights exceeding 200.00 cm.

The aboveground dry weight reflects the dry matter accumulation of barnyardgrass. *E. zelayensis* had the highest aboveground dry weight at 233.33 g, followed by *E. cruspavonis* (204.67 g), with no significant difference between these two species (Table 1). *E. colona*, *E. glabrescens*, *E. caudate*, *E. crusgalli*, and *E. mitis* showed no significant differences in dry weight, ranging from 120 to 160 g per plant. *E. oryzoides* had the lowest dry weight of only 49.00 g, which was significantly lower than that of the other barnyardgrass species. The dry weight of *E. cruspavonis* was 4.2 times higher than that of *E. oryzoides*.

## 3. Discussion

Ecotypes are defined as plants that are genetically adapted to their specific habitats while biotypes are defined as plants within ecotypes that exhibit random genetic variations [20]. Different ecotypes of barnyardgrass show morphological variations due to geographical differences [21]. This diversity in barnyardgrass weed ecotypes may contribute to their adaptation to selective pressures imposed by different management practices. Additionally, the growth habits of barnyardgrass are strongly influenced by environmental conditions, including soil conditions, fertility levels, and cultivation practices, which can affect certain morphological characteristics of barnyardgrass [22,23].

### 3.1. 1000-Grain Weight and Spikelet Number Per Panicle

The 1000-grain weight is a crucial factor influencing grain yield and is determined by the length, width, and thickness of the grains. The results revealed substantial variations in 1000-grain weight among different barnyardgrass species, with *E. oryzoides* (3.12 g) exhibiting the highest 1000-grain weight and *E. colona* (0.90 g) the lowest. These findings suggest that *E. oryzoides* outperforms *E. colona* in terms of seed productivity and grain plumpness. The quality of the seeds has a significant impact on the depth of emergence [24]. Seeds with more carbohydrate reserves have a greater potential to emerge from deeper soil levels [25]. For example, rice seeds, with an average weight of approximately 25 mg per seed, exceed *E. crusgalli* seeds, with an average weight of approximately 1.6 mg, in effectively emerging from deeper soil layers [26]. In *E. crusgalli*, seedling emergence from seeds placed on the soil declined significantly with greater planting depth but optimal emergence was observed at a depth of only 2 cm [27]. In *E. colona*, compared to the germination rates (80%) observed when sown on the soil surface, burial depths of 1.4 cm led to a 50% reduction in seedling emergence [28]. Additional studies are needed to determine the association between 1000-grain weight variations among barnyardgrass species and germination depth. Germination efficiency significantly impacts the population size and fitness [29]. The seed germination process involves complex physiological transformations that include carbohydrates, lipids, and proteins [30,31,32]. Previous studies have found a negative correlation between the 1000-grain weight and grain protein content but a positive correlation between 1000-grain weight and carbohydrate content [33]. There has been limited research on the impact of significant variations in 1000-grain weight among barnyardgrass species on seed germination. Future investigations could focus on exploring the relationships between 1000-grain weight, grain starch, lipid content, and germination characteristics in barnyardgrass species to gain a deeper understanding of germination biology.

Spikelet quantity per panicle is an important measure of the production yield, with the reproductive coefficient represented by the product of spikelet number and effective panicle number providing a rough assessment of reproductive capacity. The highest number of spikelets was found in *E. glabrescens*;its spikelets had a mean grain number of 940 compared to *E. crusgalli* which has only 278—a difference of 3.4-fold. *E. zelayensis*, *E. elongata*, and *E*. *glabrescens* exhibited the highest reproductive coefficients in the second sowing condition, indicating their capacity to generate a larger number of seeds. However, there appears to be less of a correlation between having a high reproductive capability and occurrence in the field. The frequently occurring species of barnyardgrass in Chinese rice fields are *E. crusgalli*, *E. colona*, and *E. glabrescens* [8]. This could be related to differences in the ability of various barnyardgrass species to adapt to their habitats as well as to variability in how sensitive these species are to herbicides and local cultivation systems [34].

### 3.2. The Biological Traits of Barnyardgrass Exhibit Optimal Performance in the Second Sowing Period

The tiller count, spikelet number, plant height, and other indicators are commonly employed to assess the ecological fitness of barnyardgrass [35,36]. In our study, the eight barnyardgrass species exhibited the maximum number of tillers and panicles during the second sowing condition, suggesting their optimal fitness during this specific sowing period. The second sowing time, which starts around 7 June, corresponds to the direct seeding period of rice in Jiangsu Province. This indicated that when barnyardgrass and rice occurred synchronously, the potential crop damage caused by barnyardgrass might be the most severe. Deviating from this synchronization by seeding too early or too late may reduce crop yield losses. This discovery has practical consequences for guiding herbicide application timing. Furthermore, none of the barnyardgrass species grew taller than 160.00 cm in the pot experiment. Under the same sowing conditions, various barnyardgrass species exhibited significant differences in plant height. Delayed sowing resulted in an increased number of leaves on the main stem for most barnyardgrass species. There is a known association between the number of leaves on the main stem and the formation of tillers in rice [37], with the flag leaf playing a crucial role in yield regulation. Further research is needed to investigate whether similar patterns exist in barnyardgrass.

### 3.3. The Length of the Barnyardgrass Growth Period Is Shortened with a Delayed Sowing Period

The right timing is essential for weed management and control strategies must be established in accordance with weed developmental stages. Predicting weed phenological development is, therefore, highly significant from a practical standpoint [38]. Phenology plays a crucial role in determining the outcome of crop–weed competition, with temperature and photoperiod being the two key environmental factors influencing phenological development [39,40,41]. Barnyardgrass is typically categorized as a short-day plant and a photoperiod of 9–13 h stimulates its rapid transition to reproductive growth [42]. The duration of daylight varied differently among the three sowing periods and the reduction in photoperiod may contribute to the shortened whole growth period. According to the accumulated temperature theory, under the premise of meeting other environmental conditions, within a certain temperature range there exists a positive correlation between temperature and the developmental rate of living organisms. The effective accumulated temperature required during different growth stages of the same species remains relatively stable. It is reasonable to assume that the total effective accumulated temperature for the same barnyardgrass species during the three sowing periods was similar. A shortened growth period in the same barnyardgrass species is likely attributed to achieving the required accumulated temperature more efficiently within a shorter number of days.

### 3.4. Photosynthetic Competition between Rice and Barnyardgrass Is Highly Asymmetric

All barnyardgrass species had significantly higher net photosynthetic rates than rice, with *E. crusgalli* having the greatest net photosynthetic rate, resulting in the most severe suppression of rice. Furthermore, competition with barnyardgrass also leads to a reduction in rice Rubisco activity, ROA (root oxidation activity), and Z + ZR (zeatin + zeati riboside) concentration [43]. The combined effect of the above factors results in substantial yield loss in rice during later stages. The greater height and biomass accumulation of barnyardgrass contribute significantly to this phenomenon. Certain barnyardgrass species can reach heights of nearly 200.00 cm while normal rice plants do not exceed 100.00 cm. As a result of the enormous height differences, barnyardgrass can occupy more aboveground space, shading rice plants and limiting their access to sufficient sunshine. Genomic research has indicated that barnyardgrass is capable of secreting the secondary metabolite DIMBOA through its root system, which significantly inhibits rice growth via allelopathic interactions [44].

More internodes result in greater plant height, with *E. crusgalli* having 212.67 cm of plant height corresponding to an average of 12 internodes, while *E. oryzoides* has 6 fewer internodes and a corresponding plant height of only 148.00 cm. In terms of dry matter accumulation, *E. cruspavonis* and *E. zelayensis* had the highest aboveground dry weights, exceeding 200 g per plant. The lowest dry biomass was observed in *E. oryzoides*, at only 49.00 g, which was 4.76 times lower than that in *E. mitis*. Tillering capacity is an indicator that reflects the competitive attributes of barnyardgrass. The field tiller count of barnyardgrass (Table 1) was considerably lower than that in the pot experiments (Figure 2) while the plant height was clearly greater than that in the pot experiments (Figure 3). This is primarily due to the limited tillering capacity of barnyard grass under conditions of restricted resources.

## 4. Materials and Methods

### 4.1. Plant Materials and Growth Conditions

Barnyardgrass seeds were collected from paddy fields in northeastern China for *E. oryzoides* while seeds for another seven species, including *E. cruspavonis*, *E. colona*, *E. glabrescens*, *E. caudate*, *E. crusgalli*, *E. zelayensis*, and *E. mitis*, were collected from paddy fields in Jiangsu Province. Field-collected barnyardgrass seeds were dried and stored in a cabinet at 22 °C. The experiment was conducted at the agricultural experimental station of Jiangsu Academy of Agricultural Sciences (32°18′ N, 118°52′ E) in Nanjing, Jiangsu Province, China, from April to November in 2022. The experiment was conducted in an outdoor experimental field.

Before sowing, the seeds were dried at 45 °C and then disinfected by immersing them in a 0.5% sodium hypochlorite solution for 10 min. The seeds of different barnyardgrass species were sown separately into plastic cups filled with potting soil, with one seed per cup and at least three replicates. When the barnyardgrass seedlings reached the 2–3 leaf stage, they were transplanted into plastic pots measuring 15 cm in diameter × 30 cm in height. The plastic pots were submerged in larger plastic containers and the water level in the larger containers was maintained at at least 5 cm above the rim of the smaller pots. The analyzed soil had a pH of 5.83, indicating its acidic nature. It also contained a significant amount of organic matter with a content of 48.00 g/kg. The available nitrogen, phosphorus, and potassium contents were 191.95 mg/kg, 164.98 mg/kg, and 115.28 mg/kg, respectively.

### 4.2. 1000-Grain Weight and Spikelet Number

After the maturity stage of barnyardgrass, mature seeds were sequentially collected and dried. Manual counts were meticulously conducted to ascertain the 100-grain weight for each distinct barnyardgrass species across six individual groups. Subsequently, the mean value of the six datasets was calculated and converted to the 1000-grain weight. When barnyardgrass reached the heading stage, a fully developed spike was selected and bagged and at least three replicates were collected. The spike was then dissected and the number of seeds was counted individually.

### 4.3. Biological Characteristics of Barnyardgrass

Barnyardgrass was sown in three batches (as shown in Table S1) to study the effects of different habitat conditions on the morphological characteristics of barnyardgrass. The three barnyardgrass sowing dates were governed by the rice sowing situation in the field. The most frequent field sowing date (rice transplanting) of rice in Jiangsu Province is in mid-June and the three sowing dates of barnyardgrass were set to be before, at the same time, and after the rice sowing date. The three sowing dates were 24 April, 7 June, and 26 June 2023. The main difference among the three sowing dates lies in the timing of transplanting into the plastic cups. After transplanting barnyardgrass into the plastic pots, the leaf age was recorded every 3 d. Once barnyardgrass started tillering, the number of tillers was recorded every 3 d. After barnyardgrass reached maturity, the plant height, number of leaves on the main stem, number of spikes, and number of tillers were determined.

### 4.4. Whole Growth Period

The whole growth period, starting from seed germination and extending to maturity, was measured in terms of the number of days. We defined the maturity stage of barnyardgrass as the point at which over 80% of its spikelets had completed development, seeds are easily shed, plant tillering and spikelet numbers cease to change, and the stems and leaves become withered and yellow. The complete growth period was recorded for barnyardgrass in each of the three sowing periods.

### 4.5. Field Competition Experiment

The rice variety used in the experiment was Nanjing 9108, a major cultivated japonica rice variety in Jiangsu Province. The barnyardgrass used in this experiment is the same material as the barnyardgrass sown on the second sowing date described in Section 4.3. Rice and eight barnyardgrass species were first grown in trays and when they reached the 2–3 leaf stage they were transplanted simultaneously. One barnyardgrass seedling was placed in the center of every four rice seedlings, with each species of barnyardgrass considered one treatment. There were a total of 8 treatments, each with 6 replicates. The spacing between adjacent rice plants was 20 cm. An LI-6400 XT portable photosynthesis system (LI-COR, Lincoln, NE, USA) was used to measure the net photosynthetic rate of barnyardgrass and rice during the tillering stage of barnyardgrass. When the eight ecotypes of barnyard grass reached maturity, various parameters were determined, including the plant height, aboveground dry weight, number of spikes, and number of internodes on the main stem.

### 4.6. Data Analysis

Data were exported to Excel 2016 for processing. The data were checked for their homogeneity of variances and normality of distribution and then subjected to analysis of variance (ANOVA) and a Duncan’s multiple range test in SPSS software (ver. 25.0; SPSS Inc., Chicago, IL, USA) at the 0.05 significance level. Origin software (ver. 2019b; OriginLab Corp., Wellesley Hills, MA, USA) was used for plotting. Each sample contained at least 3 biological and 3 technical replicates.

## 5. Conclusions

A biological characterization and field competition assessment of eight barnyardgrass species that commonly occur in rice fields in China was conducted in this study. The results demonstrated significant differences in 1000-grain weight and the ˙number of grains per spikelet among the barnyardgrass species. *E. oryzoides* had a 1000-grain weight of 3.13 g, while that of *E. colona* was only 0.9 g. *E. glabrescens* had the highest grain number at 940 grains/spikelet and *E. colona* had the lowest at only 277 grains/spikelet. These differences can greatly affect the seed germination characteristics and barnyardgrass seed yield, either directly or indirectly. With delayed sowing dates, distinct barnyardgrass species demonstrated notable variations in their biological traits. With the exception of *E. caudate*, the remaining barnyardgrass species exhibited a significant reduction in growth duration with delayed the sowing dates. This may be primarily attributed to shorter photoperiods and higher effective accumulated temperatures. Compared to those in the pot experiments, the barnyardgrass species had much fewer tillers under the field competition experiment but their plant height increased significantly. Additionally, the field competition experiment demonstrated extremely asymmetric competition between barnyardgrass and rice in terms of photosynthesis. In the presence of barnyardgrass interference, the photosynthetic rates of rice were on average lower than 50% of those of the control species. This asymmetric competition directly led to severe rice yield losses in the later stages. These findings will contribute to a deeper understanding of the interspecific differences in biological traits of barnyardgrass and provide a theoretical basis for the scientific management of barnyardgrass in field settings.

## Figures and Tables

**Table 1:** Differences in the biological characteristics of different barnyardgrass species under field competition.

*Echinochloa* spp.	Tiller (No.)	Height (cm)	Node No. of the Main Stem	Shoot Dry Weight (g)
*E. cruspavonis*	12.67 ± 0.58 e	200.67 ± 10.97 b	8.00 ± 0.58 c	204.67 ± 23.46 a
*E. oryzoides*	14.67 ± 0.58 de	148.00 ± 6.24 d	6.00 ± 0.58 d	49.00 ± 14.42 c
*E. colona*	48.67 ± 5.51 a	171.67 ± 10.41 c	9.00 ± 0.58 c	154.67 ± 19.40 b
*E. glabrescens*	32.00 ± 3.61 b	183.33 ± 2.89 c	9.00 ± 0.58 c	140.67 ± 19.22 b
*E. caudate*	22.33 ± 2.52 c	217.67 ± 2.52 a	9.00 ± 0.58 c	141.00 ± 25.51 b
*E. zelayensis*	32.00 ± 4.00 b	198.00 ± 2.65 b	9.00 ± 0.00 c	233.33 ± 6.66 a
*E. crusgalli*	19.00 ± 3.46 cd	212.67 ± 14.19 ab	12.00 ± 1.53 a	165.00 ± 28.62 b
*E. mitis*	20.33 ± 2.52 cd	209.00 ± 7.94 ab	11.00 ± 0.58 b	128.00 ± 15.13 b

The results are expressed as the mean values ± standard deviations and different letters within a column indicate significant differences at the 0.05 significance level. Number of nodes on the main stem: rounding to two decimal places for the mean value was performed to obtain practical numbers (rounding down for values < 0.50 and rounding up for values ≥ 0.50).

## Data Availability

The data is contained within the manuscript and Supplementary Materials.

## Supplementary Materials

The following supporting information can be downloaded at: https://www.mdpi.com/article/10.3390/plants12173085/s1, Table S1: Three batches of barnyardgrass sowing dates.

## References

[1] Fang J., He Z., Liu T., Li J., Dong L. (2020). A novel mutation Asp-2078-Glu in ACCase confers resistance to ACCase herbicides in barnyardgrass (*Echinochloa crus-galli*). Pestic. Biochem. Physiol..

[2] Kaya Altop E., Mennan H. (2010). Genetic and morphologic diversity of *Echinochloa crus-galli* populations from different origins. Phytoparasitica.

[3] Yang X., Han H., Cao J., Li Y., Yu Q., Powles S.B. (2021). Exploring quinclorac resistance mechanisms in *Echinochloa crus-pavonis* from China. Pest Manag. Sci..

[4] Holm L.G., Plucknett D.L., Pancho J.V., Herberger J.P. (1977). The World’s Worst Weeds.

[5] Zhang Z., Cao J., Gu T., Yang X., Peng Q., Bai L., Li Y. (2021). Co-planted barnyardgrass reduces rice yield by inhibiting plant above- and belowground-growth during post-heading stages. Crop J..

[6] Peng Q., Han H., Yang X., Bai L., Yu Q., Stephen B.P. (2019). Quinclorac Resistance in *Echinochloa crus-galli* from China. Rice Sci..

[7] Zhu J., Wang J., DiTommaso A., Zhang C., Zheng G., Liang W., Islam F., Yang C., Chen X., Zhou W. (2020). Weed research status, challenges, and opportunities in China. Crop Prot..

[8] Chen G., Tang W., Li J., Lu Y., Dong L. (2019). Distribution characteristics of *Echinocloa* species in rice fields in China: A case survey on 73 sites from nine provincial administrative regions. Chin. J. Rice Sci..

[9] Lu Y., Liu D., Yu L., Liu D., Guo S. (2014). Classification and diversity of *Echinochloa* in paddy fields of main agricultural regions in China. Plant Sci. J..

[10] Smith R.J. (1988). Weed thresholds in southern US rice, Oryza sativa. Weed Technol..

[11] Zhang Z., Gu T., Zhao B., Yang X., Peng Q., Li Y., Bai L. (2017). Effects of common *Echinochloa* varieties on grain yield and grain quality of rice. Field Crop. Res..

[12] Ottis B.V., Talbert R.E. (2017). Barnyardgrass (*Echinochloa crus-galli*) Control and Rice Density Effects on Rice Yield Components. Weed Technol..

[13] Chauhan B.S., Johnson D.E. (2010). Responses of Rice Flatsedge (*Cyperus iria*) and Barnyardgrass (*Echinochloa crus-galli*) to Rice Interference. Weed Sci..

[14] Tang J., Xie J., Chen X., Yu L. (2009). Can rice genetic diversity reduce *Echinochloa crus-galliinfestation*?. Weed Res..

[15] Wu H., Walker S.R., Osten V.A., Robinson G. (2010). Competition of sorghum cultivars and densities with Japanese millet (*Echinochloa esculenta*). Weed Biol. Manag..

[16] de Vida F.B.P., Laca E.A., Mackill D.J., Fernández G.M., Fischer A.J. (2006). Relating rice traits to weed competitiveness and yield: A path analysis. Weed Sci..

[17] Moshatati A., Gharineh M. (2012). Effect of grain weight on germination and seed vigor of wheat. Int. J. Agric. Crop Sci..

[18] Norris R.F. (1992). Relationship between inflorescence size and seed production in barnyardgrass (*Echinochloa crus-galli*). Weed Sci..

[19] Swanton C.J., Huang J.Z., Shrestha A., Tollenaar M., Deen W., Rahimian H. (2000). Effects of temperature and photoperiod on the phenological development of barnyardgrass. Agron. J..

[20] Klingaman T.E., Oliver L.R. (1996). Existence of ecotypes among populations of entireleaf morningglory (*Ipomoea hederacea var integriuscula*). Weed Sci..

[21] Sastroutomo S.K.S., Juraimi A.S., Kadir J.B., Napis S., Tasrif A. (2004). Morphological variation of the ecotypes of *echinochloa crus-galli var crus-galli* (L). *Beauv* (barnyard grass: Poaceae) in malaysia and indonesia. Biotropia SEA J. Trop. Biol..

[22] Yabuno T. Biology of *Echinochloa* species. Proceedings of the Conference on Weed Control in Rice.

[23] Lopez-Martinez N., Salva A.P., Finch R.P., Marshall G., De Prado R. (1999). Molecular markers indicate intraspecific variation in the control of *Echinochloa* spp. with quinclorac. Weed Sci..

[24] Chauhan B.S., Johnson D.E. (2011). Ecological studies on *Echinochloa crus-galli* and the implications for weed management in direct-seeded rice. Crop Prot..

[25] Baskin C.C., Baskin J.M. (1998). Seeds: Ecology, Biogeography, and, Evolution of Dormancy and Germination.

[26] Moon B.-C., Park S.-T., Kim S.-C., Kwon S.-J., Mortimer A.M., Piggin C. (1999). Weed emergence as affected by burying depth and water management. Korean J. Med. Crop Sci..

[27] Sadeghloo A., Asghari J., Ghaderi-Far F. (2013). Seed germination and seedling emergence of velvetleaf (*Abutilon theophrasti*) and barnyardgrass (*Echinochloa crus-galli*). Planta Daninha.

[28] Zhang Z.C., Wang H.C., Cao J.J., Li G., Chauhan B.S. (2023). Seed biology of alkali barnyardgrass (*Echinochloa crus-galli var. zelayensis*) and junglerice (*Echinochloa colona*) for improved management in direct-seeded rice. Weed Sci..

[29] Lai L., Tian Y., Wang Y., Zhao X., Jiang L., Baskin J.M., Baskin C.C., Zheng Y. (2015). Distribution of three congeneric shrub species along an aridity gradient is related to seed germination and seedling emergence. AoB Plants.

[30] Hager A.-S., Mäkinen O.E., Arendt E.K. (2014). Amylolytic activities and starch reserve mobilization during the germination of quinoa. Eur. Food Res. Technol..

[31] Alhadi Fatima A., Al-Asbahi Adnan A.S., Alhammadi Arif S.A., Abdullah Qais A.A. (2012). The effects of free amino acids profiles on seeds germination/dormancy and seedlings development of two genetically different cultivars of Yemeni Pomegranates. J. Stress Physiol. Biochem..

[32] Suda C.N., Giorgini J.F. (2000). Seed reserve composition and mobilization during germination and initial seedling development of *Euphorbia heterophylla*. Rev. Bras. Fisiol. Veg..

[33] Abdipour M., Ebrahimi M., Izadi-Darbandi A., Mastrangelo A.M., Najafian G., Arshad Y., Mirniyam G. (2016). Association between Grain Size and Shape and Quality Traits, and Path Analysis of Thousand Grain Weight in Iranian Bread Wheat Landraces from Different Geographic Regions. Not. Bot. Horti Agrobot. Cluj-Napoca.

[34] Wu D., Shen E., Jiang B., Feng Y., Tang W., Lao S., Jia L., Lin H.Y., Xie L., Weng X. (2022). Genomic insights into the evolution of *Echinochloa* species as weed and orphan crop. Nat. Commun..

[35] Bagavathiannan M.V., Norsworthy J.K., Jha P., Smith K. (2017). Does Resistance to Propanil or Clomazone Alter the Growth and Competitive Abilities of Barnyardgrass (*Echinochloa crus-galli*)?. Weed Sci..

[36] Panozzo S., Scarabel L., Rosan V., Sattin M. (2017). A New Ala-122-Asn Amino Acid Change Confers Decreased Fitness to ALS-Resistant *Echinochloa crus-galli*. Front. Plant Sci..

[37] Counce P.A., Siebenmorgen T.J., Poag M.A., Holloway G.E., Kocher M.F., Lu R.F. (1996). Panicle emergence of tiller types and grain yield of tiller order for direct-seeded rice cultivars. Field Crop. Res..

[38] Necajeva J., Royo-Esnal A., Loddo D., Jensen P., Taab A., Synowiec A., Uludag A., Uremis I., Murdoch A., Bochenek A. (2022). Phenological development of barnyard grass plants originating from different geographical locations. Agron. J..

[39] Major D.J., Kiniry J.R. (1991). Predicting Daylength Effects on Phenological Processes.

[40] Patterson D.T. (1993). Effects of Temperature and Photoperiod on Growth and Development of Sicklepod (*Cassia obtusifolia*). Weed Sci..

[41] Ghersa C.M., Holt J.S. (1995). Using phenology prediction in weed management: A review. Weed Res..

[42] Bajwa A.A., Jabran K., Shahid M., Ali H.H., Chauhan B.S. (2015). Eco-biology and management of *Echinochloa crus-galli*. Crop Prot..

[43] Wang X.L., Zhang Z.Y., Xu X.M., Li G. (2019). The density of barnyard grass affects photosynthesis and physiological characteristics of rice. Photosynthetica.

[44] Guo L., Qiu J., Ye C., Jin G., Mao L., Zhang H., Yang X., Peng Q., Wang Y., Jia L. (2017). *Echinochloa crus-galli* genome analysis provides insight into its adaptation and invasiveness as a weed. Nat. Commun..

