# Supplementary material for: Inter-Species Investigation of Biological Traits among Eight Echinochloa Species
Source: Plants (Basel). 2023 Aug 28;12(17):3085. doi: 10.3390/plants12173085 (PMC10489896; doi:10.3390/plants12173085)
Supplement: Supplementary file 1 [file plants-12-03085-s001.zip › plants-2547576-supplementary.pdf]

Supplementary Materials

Table S1. Three batches of barnyardgrass sowing dates.

	Germination in the incubator (30/25°C)	Transplanting plastic cups	Plastic bucket
**The 1st sowing**	4.20 – 4.23	4.24 – 5.10	5.11 – maturity
**The 2nd sowing**	6.3 – 6.6	6.7 – 6.13	6.14 – maturity
**The 3rd sowing**	——	6.26 – 7.5	7.6 – maturity

This experiment was conducted in 2022. Similar to "4.20–4.23", this notation indicates a range from one specific day of the month to another specific day of the month. The rice seeds are first germinated in a incubator and after germination, they will be transplanted into plastic cups for growth in an outdoor environment. The main difference among the three sowing dates lies in the timing of transplanting into the plastic cups.
